# Connectedness to Nature and to Humanity: their association and personality correlates

**DOI:** 10.3389/fpsyg.2015.01003

**Published:** 2015-07-21

**Authors:** Kibeom Lee, Michael C. Ashton, Julie Choi, Kayla Zachariassen

**Affiliations:** ^1^Department of Psychology, University of CalgaryCalgary, AB, Canada; ^2^Department of Psychology, Brock UniversitySt. Catharines, ON, Canada

**Keywords:** identification with all humanity, Connectedness to Nature, honesty–humility, openness to experience, HEXACO

## Abstract

People differ in the extent to which they identify with humans beyond their ingroup and with non-human living things. We refer to the former as the Connectedness to Humanity (CH) and to the latter as the Connectedness to Nature (CN). In a sample of 324 undergraduate students, CH and CN were operationalized using the Identification with All Humanity Scale (McFarland et al., 2012) and the CN Scale (Mayer and Frantz, 2004), respectively. These variables correlated moderately with each other (*r* = 0.44) and shared Openness to Experience and Honesty–Humility as their primary personality correlates. CN was found to play an important role in mediating the relationships between the two personality variables and some specific pro-environmental/pro-animal attitudes and ecological behaviors.

## Introduction

People differ in the extent to which they feel that they are connected to other individuals. Such feelings of connectedness may be formed around smaller groups such as families and local communities, or around larger entities such as national, racial or ethnic, and religious groups. However, some people transcend such identifiable group boundaries and feel connectedness even to persons beyond those boundaries. Moreover, feelings of connectedness can even be extended to the natural world encompassing non-human beings, particularly animals. In the present research, we examine the constructs of Connectedness to Humanity (CH) and Connectedness to Nature (CN) in terms of their relations with each other as well as their common personality correlates and their links with some specific pro-environmental and pro-animal attitudes and ecological behaviors. We begin by reviewing some empirical and conceptual work on these constructs.

## Connectedness to Nature and to Humanity

### Connectedness to Nature

Several constructs in environmental psychology are characterized by a sense of belongingness to the natural world, or of viewing oneself as a part of nature (see Schultz, 2002; Mayer and Frantz, 2004; Nisbet et al., 2009). Brügger et al. (2011) and Tam (2013) examined several measures assessing these constructs. In both studies, those measures generally showed a strong level of convergence, and thereby it was concluded that these measures “can be considered as markers of a common construct” (Tam, 2013, p. 64), and in this article we refer to this construct as CN. CN has been suggested to be one of the most important constructs in shaping individuals’ attitudes and behaviors related to environmentalism (Stern, 2000). Consistent with this, Tam (2013) reported that measures of CN correlated moderately strongly with environmental attitudes (*r*s = from 0.40 s to 0.50 s) and ecological behaviors (*r*s = 0.30 s).

### Connectedness to Humanity

A tendency to identify with all humanity (i.e., beyond one’s own group boundaries) has also been conceptualized by several researchers. For example, Buchan et al. (2011) showed that social identification can be meaningfully applied to the world community and that such an inclusive social identity was related to altruistic behaviors toward people considered outgroup members. More recently, a similar construct involving global identity has also been investigated within environmental psychology. For example, Der-Karabetian et al. (2014) measured a construct that they called “Global Belonging” (e.g., think of myself as a citizen of the world; feel that I am related to everyone in the world as if they were my family), and showed its relevance to pro-environmental behaviors. Devine-Wright et al. (2015) investigated a similar concept known as global placement attachment. It was found that those who express stronger global attachment than national attachment are more likely to believe that humans have contributed to climate change and that actions are needed to limit its effects. One intensively validated measures of CH is the construct known Identification with All Humanity (IWAH; McFarland et al., 2012). IWAH is defined as a tendency to feel care and concern for all human beings, regardless of racial, religious, or national boundaries. People high in IWAH are characterized by “a sense of belongingness to one human family” (McFarland et al., 2013, p. 194), and by the transcendence of group boundaries between broad human collectives. In the present research CH was assessed with the IWAH scale.

### Relation between Connectedness to Nature and to Humanity

We suggest that the two kinds of constructs described above can be considered as sharing a sense of unity or oneness, to two different entities: Nature and Humanity. Despite the different nature of the targets that one identifies with, we expect these two constructs to correlate positively with one another, partly because they are likely to share some common personality correlates.

Before discussing the personality correlates of the CN and CH, we discuss some previous studies investigating the relationships among self-identity variables similar to CN and CH. Leary et al. (2008) suggested that self-identity can be extended to broader categories of people, animals, and non-living objects. The researchers called this form of identity *allo-inclusive identity*, and developed a measure assessing the construct. Each statement asks people to indicate the extent to which they feel a connection between them and a group of other people (e.g., the best friends of your sex, the average American, homeless person on the street, your family, etc.) or a thing (a dog, a tree, the Earth, etc.). The Allo-Inclusive Scale (AIS) consists of the two subscales—AI-Natural World and AI-People—that have some resemblance to CN and to CH, respectively. As we would expect, these two subscales were found to correlate positively with each other (*r* = 0.35).

We should note, however, that while AI-Natural World is closely related to CN, AI-People does not correspond squarely to CH, because most of the referenced “people” in the scale are friends, family members, and strangers within one’s own community and country. As such, AI-People measures a general sense of feeling connected to people, rather than the transcendence of broad human collectives such as community, country, and so on. Moreover, AI-People tends to show a pattern of relationships with personality variables different from that shown by markers of CH. For example, as we will discuss later in this manuscript, Openness to Experience is suggested and found to correlate positively with CH (McFarland et al., 2012), but it showed a near-zero correlation with the AI-People scale in the Leary et al. (2008) study.

Arnocky et al. (2007, p. 256) also examined a construct named metapersonal self-construal, which “involves the perception of the self as having a deep interconnection with all forms of life.” The authors investigated the construct in relation to Markus and Kitayama’s (1991) well-known constructs of independent and interdependent self-construal. Although the authors showed that the three constructs are positively correlated with each other (Stronik and DeCicco, unpublished, cited in Arnocky et al., 2007), these results have only indirect implications to the question posed in the present research, for at least two reasons. First, metapersonal self-construal is conceptually very similar to CN, but it also includes some other aspects such as spiritual sensibility and existential meaningfulness (Leary et al., 2008). Second, the two self-construal constructs are primarily about individual differences in defining the self interdependently with others or independently from others, but “others” in these constructs are generally people who belong to one’s ingroup, and therefore the self-construal constructs do not have a strong conceptual link to CH.

As seen from the above review, the constructs investigated by Arnocky et al. (2007) and Leary et al. (2008) differ from the CH construct examined in the present research. Specifically, the former constructs do not involve the transcendence of human ingroup boundaries, which is a defining characteristic of CH. It is this element of transcendence that aligns the CH construct with the construct of CN, which is characterized by the transcendence of the boundary between human and non-human living things. That is, the two ostensibly distinct constructs, CH and CN, share a common characteristic defined by the fuzziness versus sharpness of category boundaries. As we discuss in the next section, this common characteristic involving fuzzy versus sharp boundaries leads to some hypotheses regarding personality correlates of CH and CN. Below, we discuss some personality traits that are expected to be associated with both CH and CN, and suggest that these common personality traits account for part of the positive relationship between CN and CH. To test this hypothesis, we examined in this study the locations of the CN and CH constructs within the six-dimensional HEXACO model of personality structure, and we tried to explain the CH—CN relationship in terms of the proposed common personality traits.

## Common Personality Correlates of Connectedness to Nature and to Humanity

The HEXACO model of personality structure is based on findings from lexical studies of personality structure, which involve factor analyses of ratings on personality-descriptive adjectives. Recent cross-language reviews of these studies suggest that six lexical personality factors are widely replicable, rather than only five as previously thought (Ashton and Lee, 2007; Lee and Ashton, 2008). These findings suggest that a comprehensive model of personality should include six basic dimensions. Of the six HEXACO personality factors, three (Extraversion, Conscientiousness, and Openness to Experience) are very similar to the same-named factors of the Big Five, but Emotionality and Agreeableness are somewhat different from Neuroticism and Agreeableness of the Big Five. Specifically, these two HEXACO factors can be viewed roughly as rotated variants of the corresponding Big Five factors (see Ashton et al., 2014a). The HEXACO Honesty–Humility factor has no direct counterpart in the Big Five, with most of its defining traits being peripherally related to Big Five Agreeableness. Thus, the variances of Big Five Agreeableness and Neuroticism are redistributed in the HEXACO model into Honesty–Humility, Agreeableness, and Emotionality, which also contain some non-Big Five variance. These latter three HEXACO constructs provide a more differentiated representation of the traits underlying altruistic tendencies (Ashton et al., 2014a). As we suggest below, such differentiated conceptualizations are particularly helpful in delineating some aspects of CN and CH. We suggest that Honesty–Humility and Openness to Experiences are the primary personality correlates both of CH and of CN, and below we provide some conceptual rationales and empirical findings regarding these relationships.

### Openness to Experience

The Openness to Experience dimension of personality is related to a tendency to draw a fuzzier distinction between ingroups and outgroups. For example, people high in Openness tend to show a more favorable attitude than do people low in Openness toward people with different sexual orientations (Cullen et al., 2002) and different ethnic backgrounds (Flynn, 2005). High-Openness individuals’ receptiveness to outgroup members might be explained by their desire to prefer novelty and variety over conventionality and uniformity. At a broader level, McCrae (1994) has also suggested that the Openness to Experience factor is related to Hartmann’s (1991) construct of boundaries in the mind (which captures the permeability of the mental divisions between the contents of consciousness), and reported a fairly strong correlation between Openness to Experience and permeable mental boundaries (see also Van Hiel and Mervielde, 2004).

We suggest that the sharpness of psychological boundaries between ingroups and outgroups may influence both CN and CH. For example, CH is characterized by a tendency to see all humanity as one family, which therefore suggests a lack of perceived boundaries among people of different race, religion, and nationality. Analogously, CN is characterized by a lack of perceived boundaries between humans and other living things, as demonstrated by such items included in the CN Scale (CNS; Mayer and Frantz, 2004) as, “I often feel a sense of oneness with the natural world around me.” We therefore expect measures of CH and CN to show a moderately strong positive correlation with each other, and we further expect that Openness to Experience will explain some substantial part of this covariation.

Strong associations of Openness to Experience with CH or with CN have been reported in previous studies. McFarland et al. (2012) found Openness to Experience to be the strongest personality correlate of the IWAH among the HEXACO personality factors, with correlations approaching 0.40. Markowitz et al. (2012) also reported that Openness to Experience as measured by the Big Five Inventory correlated 0.45 with the CNS. Furthermore, some other constructs that resemble CH and CN also tend to show positive correlations with Openness to Experience. For example, Piedmont (1999, p. 995) reported that a “Universality” scale—assessing feelings that all life is interconnected and of an emotional bond with all of humanity—correlated more strongly with the NEO-PI-R Openness to Experience scale than with any other domain scales in the inventory. Thus, as described above, conceptual similarities and some empirical evidence suggest that CH and CN should both be associated with the Openness to Experience factor of personality (see also Tam, 2013).

### Honesty–Humility

Honesty–Humility is defined as a tendency to cooperate with (or not to exploit) others even when one could successfully exploit them (Ashton and Lee, 2007). Feeling connected to strangers beyond one’s own ingroup boundaries (family, community, or country) generally involves moral concerns about the welfare of the strangers, which characterizes people high in Honesty–Humility. For people lacking such moral concerns, strangers or outgroup members are perceived to be an easy “target” for exploitation because strangers often do not have a means to retaliate or because community norms do not necessarily protect outsiders. People having a fuzzier distinction between ingroup and outgroup boundaries (i.e., high in CH), therefore, would not perceive outgroup members as targets for exploitation, and would instead tend to deal fairly with strangers and outgroup members (cf. Cohen et al., 2006). Consistent with this suggestion, McFarland et al. (2012) found that Honesty–Humility (and also the Agreeableness factor in the HEXACO model) correlated in the 0.20 s or 0.30 s with IWAH; only Openness to Experience showed stronger correlations with IWAH.

With respect to CN, some researchers have suggested that prosocial motivations may underlie people’s attitudes about the environment such as a sense of unity to the natural world (Heberlein, 1972; Stern, 2000). This idea is based upon the premise that the environment is a public good, the misuse of which produces negative externalities that affect all other people. Therefore, people showing an inherent concern about the natural world (e.g., people high in CN) are likely to have prosocial personality characteristics such as Honesty–Humility. Interestingly, Honesty–Humility has been found to be the personality factor that can best predict altruistic behaviors displayed in a public goods game (Hilbig et al., 2012). As such, we expect that Honesty–Humility should positively correlate with CN (and with other specific attitudes and behaviors influenced by CN).

Despite such a plausible link between pro-social personality traits and CN, previous studies have produced mixed findings regarding whether pro-social personality traits (e.g., Honesty–Humility and Big Five Agreeableness) are implicated in one’s sense of belongingness to nature. Whereas some studies have reported that Honesty–Humility or Big Five Agreeableness are positively associated with CN or variables strongly influenced by it (Nisbet et al., 2009; Hirsh, 2010; Hilbig et al., 2013; Brick and Lewis, in press), other studies did not find such relationships. Specifically, in two US samples, Markowitz et al. (2012) reported near-zero correlations of the CNS and other pro-environmental behaviors/attitudes with measures of Big Five Agreeableness and of Honesty–Humility. A near-zero correlation was also obtained between Big Five Agreeableness and the Allo-Inclusive: Nature scale, which is similar to CN (*N* = 148, Leary et al., 2008).

## Pro-Environmental, Pro-Animal Attitudes, and Ecological Behavior

As described above, the present study is intended to examine the relations between CN and CH and their common personality bases. An additional aim of this research is to investigate how CN and CH influence specific behaviors and attitudes toward environment and non-human animals (hereafter animals). We hypothesize that CN and CH could be proximally related to pro-environmental/pro-animal attitudes and behavior, mediating the links between personality traits and those variables.

With regards to CN, we suggest that people who believe that they are part of the natural world are inclined to protect it. Consistent with this view, previous studies have consistently reported moderately strong correlations of CN with beliefs about pro-environmental attitudes and with ecological behaviors (Schultz, 2000; Nisbet et al., 2009; Markowitz et al., 2012). In addition, people’s attitudes toward animals are likely to be influenced by the extent to which one identifies with nature more broadly. Nisbet et al. (2009) reported a correlation of 0.34 between the Nature Relatedness scale and the love of animal scale (adapted from Ray, 1982). We therefore expect CN to be significantly associated with pro-environmental and pro-animal attitudes and behaviors, and to play an important mediating role in the relations between personality characteristics (i.e., Openness to Experience and Honesty–Humility) and the attitudinal and behavioral variables.

Regarding the link between CH and variables related to pro-environmental/pro-animal attitudes, some theorists have considered altruistic values (akin to CH) as an important basis of environmental attitudes in addition to biospheric values (Stern and Dietz, 1994 for a review). According to this view, environmental attitudes and behaviors are based on altruistic concerns about other people, and engaging in environmentally protecting behaviors stems from one’s motives to benefit (or not to harm) other people (Heberlein, 1972; Stern, 2000). As previously mentioned, some studies have measured constructs very similar to CH, and the results from these studies generally support the notion that CH is positively related to pro-environmental variables. For example, Der-Karabetian et al. (2014) reported correlations in the 0.20 and 0.30 s between “Global Belonging” and sustainable behaviors in three samples from the US, China, and Taiwan. Similarly, Devine-Wright et al. (2015) found that persons with stronger global attachment (relative to national attachment) showed more concern about climate change than did persons with stronger national attachment (relative to global attachment). As such, we expect that CH partly mediates the relations of Honesty–Humility/Openness to Experience to pro-environmental/animal attitudes and ecological behaviors.

To evaluate the research questions outlined above, we tested a latent variable model in which Honesty–Humility and Openness to Experience are exogenous variables that influence CN and CH, which in turn influence one of the three specific variables, namely pro-environmental attitudes, pro-animal attitudes, and ecological behaviors.

## Summary of the Present Research

In the present research, we posit that the tendencies to feel connected to humanity and to the natural world have a common psychological basis in being characterized by a lack of sharp boundaries between oneself and (a) other human beings beyond one’s ingroups (in the case of CH) or (b) other non-human living things (in the case of CN). We aim to address several questions surrounding the two constructs in terms of their relations with each other, their common personality roots, and their impact on pro-environmental/pro-animal attitudes and behaviors. The present research is thus intended to complement findings from previous research, in several ways.

First, although CN has been investigated in term of its relations to some variables related to self-identity (Arnocky et al., 2007; Leary et al., 2008), the latter variables differ from CH as described in the present research, which is essentially identification with human beings *beyond one’s ingroup boundaries* (McFarland et al., 2012, 2013). In this study, therefore, we examined the relationship between CN and CH and their common personality correlates. Specifically, we hypothesized that the Openness to Experience and Honesty–Humility personality dimensions can partly explain the expected positive relationship between the CN and CH constructs, and that these personality factors may have an impact on pro-environmental/animal attitudes and ecological variables through their effects on CN and CH.

Second, theorists in environmental psychology have suggested altruism as one of the fundamental motives underlying pro-environmental attitudes and behaviors, and the present research revisits this issue through the construct of CH and a prosocial personality trait, namely Honesty–Humility. In this way, the present research provides some additional clarifications about the empirical link between prosocial personality traits and pro-environmental attitudes and ecological behaviors, as previous studies have produced inconsistent results.

Finally, previous studies investigating personality traits and variables related to environmentalism have relied on a single rating source, typically self-reports. This may have influenced the observed effect sizes of the relationships due to common rating source variance. In the present research we obtained both self- and observer ratings of personality, and we thereby tried to provide conservative estimates as to the extent to which personality influences the pro-environmental and pro-animal variables included in the study.

## Materials and Methods

### Participants

Participants were recruited through psychology research participant pools in a Canadian University. Interested undergraduate students were instructed to come to the laboratory sessions with a well-acquainted person (such as a friend, a romantic partner, or a relative) whom they had known for at least 1 year. Both members in each of these dyads participated in the study, with each member independently providing personality self-reports and personality observer reports (of the other dyad member) as well as self-reports of the other variables described below. (Some variables administered to this sample for other research projects are not described in the present research.) A total of 324 individuals participated (mean age = 19.7 [SD = 2.0]; 63.2 % female). This sample was part of the sample used in another published article on an unrelated topic (see Ashton et al., 2014b). This study was carried out in accordance with the recommendations of the Conjoint Faculties Research Ethics Board at the University of Calgary. Following the Boards’ recommendation, written informed consent was not obtained. However, all the information normally included in a consent form was provided in a covering letter, and the letter included a statement that “participants’ decision to complete and return this questionnaire will be interpreted as an indication of the consent to participate.”

### Measures

All the scales described below were measured on a five-point scale (1 = *Strongly Disagree*; 5 = *Strongly Agree*) unless indicated otherwise. **Table 1** shows the means, standard deviations, and internal consistency reliabilities of the scales in the present sample.

**Table 1:** Means, SD, correlations, and coefficient alphas for study variables.

	*M*	SD	1	2	3	4	5	6	7	8	9	10	11	12	13	14	15	16	17	18	19
**Self-Reports**
(1) CN (Connectedness)	3.28	0.54	(0.82)	–	–	–	–	–	–	–	–	–	–	–	–	–	–	–	–	–	–
(2) CH (IWAH)	2.97	0.71	0.44	(0.84)	–	–	–	–	–	–	–	–	–	–	–	–	–	–	–	–	–
(3) Identification with Community	3.43	0.78	0.26	0.50	(0.88)	–	–	–	–	–	–	–	–	–	–	–	–	–	–	–	–
(4) Identification with Country	3.34	0.68	0.32	0.74	0.61	(0.85)	–	–	–	–	–	–	–	–	–	–	–	–	–	–	–
(5) Animal attitudes	3.30	0.56	0.41	0.18	0.03	0.06	(0.87)	–	–	–	–	–	–	–	–	–	–	–	–	–	–
(6) Pro-environmental attitudes (NEP)	3.51	0.47	0.40	0.14	-0.09	0.02	0.42	(0.77)	–	–	–	–	–	–	–	–	–	–	–	–	–
(7) Ecological Behavior	3.20	0.41	0.37	0.27	0.12	0.18	0.19	0.31	(0.62)	–	–	–	–	–	–	–	–	–	–	–	–
(8) Honesty-Humility	3.30	0.58	0.32	0.25	0.17	0.14	0.25	0.16	0.21	(0.80)	–	–	–	–	–	–	–	–	–	–	–
(9) Emotionality	3.42	0.61	0.13	0.07	0.13	0.10	0.26	0.08	0.14	0.10	(0.83)	–	–	–	–	–	–	–	–	–	–
(10) Extraversion	3.46	0.57	0.07	0.16	0.27	0.26	-0.02	0.02	-0.10	-0.06	-0.05	(0.84)	–	–	–	–	–	–	–	–	–
(11) Agreeableness	3.02	0.61	0.16	0.18	0.15	0.13	-0.06	0.03	0.08	0.28	-0.10	0.10	(0.85)	–	–	–	–	–	–	–	–
(12) Conscientiousness	3.50	0.58	0.10	0.11	0.13	0.14	0.07	-0.01	0.15	0.07	0.13	0.18	0.14	(0.84)	–	–	–	–	–	–	–
(13) Openness to Experience	3.21	0.65	0.44	0.39	0.06	0.23	0.12	0.32	0.32	0.18	0.00	0.04	0.26	0.05	(0.82)	–	–	–	–	–	–
**Observer Reports**
(14) Honesty–Humility	3.28	0.60	0.17	0.08	0.11	0.07	0.14	0.10	0.28	0.44	0.15	-0.18	0.13	0.03	0.17	(0.84)	–	–	–	–	–
(15) Emotionality	3.29	0.58	0.16	0.12	0.15	0.12	0.25	-0.02	0.02	0.11	0.53	0.07	0.03	0.13	0.00	0.07	(0.84)	–	–	–	–
(16) Extraversion	3.51	0.59	0.01	0.10	0.12	0.15	-0.07	-0.01	-0.03	-0.06	-0.13	0.58	0.08	-0.00	0.14	-0.06	-0.07	(0.87)	–	–	–
(17) Agreeableness	3.21	0.65	0.11	0.07	0.08	0.08	-0.09	0.00	0.09	0.08	-0.02	-0.04	0.41	-0.03	0.25	0.35	-0.10	0.20	(0.89)	–	–
(18) Conscientiousness	3.52	0.62	0.07	0.07	0.12	0.09	0.07	-0.01	0.12	0.14	0.24	-0.11	0.10	0.41	0.14	0.28	0.12	0.02	0.17	(0.88)	–
(19) Openness to Experience	3.05	0.62	0.31	0.27	0.00	0.13	0.04	0.28	0.32	0.13	0.07	-0.00	0.17	0.02	0.60	0.29	0.04	0.15	0.29	0.22	(0.83)

*N = 324. CN, Connectedness to Nature; CH (IWAH), Connectedness to Humanity as measured by the Identification With All Humanity; NEP, New Environmental Paradigm. Values in the diagonal are coefficient alphas.*

#### HEXACO Personality Inventory–Revised

The 100-item version of the HEXACO-PI-R (Lee and Ashton, 2004) was used to assess self- and observer reports of personality (see http://hexaco.org for the items of the inventory).

#### Connectedness to Humanity

We used McFarland et al. (2012) IWAH scale. This scale consists of nine three-part items, each following the form of this sample item, “How much do you identify with each of the following? (a) people in my community, (b) people in my country, (c) all humans everywhere” Following McFarland et al. (2012) we calculated the mean of the responses on the third option for each individual and used it for a measure of CH (see McFarland et al., 2012, for construct validity evidence of the IWAH scale). Because the three identification variables are likely to correlate strongly with each other, McFarland et al. (2012) suggested controlling for the other two identification variables to examine the unique characteristics of the IWAH. In the present research we followed this practice.

#### Connectedness to Nature

Mayer and Frantz’s (2004) 14-item CNS was used. The scale assesses one’s feeling of belongingness to the natural world, a sample item being “I often feel a sense of oneness with the natural world around me.”

#### Pro-Environmental Attitudes and Behaviors

We included the 15-item New Environmental Paradigm (NEP) scale (Dunlap et al., 2000) to measure beliefs about humans’ interactions with the environment. Sample items include “We are approaching the limit of the number of people the earth can support” and “Humans were meant to rule over the rest of nature.”

Self-reports of pro-environmental behavior were measured using an 18-item scale developed by Kaiser et al. (2007). The scale includes behaviors that promote ecological preservation and environmental protection (“I bring empty bottles to a recycling bin” and “I contribute financially to environmental organizations”). Participants were asked to indicate how often they engage in such behaviors using a five-point scale (1 = *never*; 5 = *always*).

#### Pro-Animal Attitudes

The 29-item Animal Attitudes Scale of Herzog et al. (1991) was used to assess participants’ opinions on the treatment and consumption of animals. High overall scores on the scale indicated positive and protective attitudes toward animals. The scale included statements about hunting, farming practices, and the use of animals for human entertainment. Sample items include “I do not think that there is anything wrong with using animals in medical research” (R) and “I sometimes get upset when I see wild animals in cages at zoos.”

## Results

**Table 1** shows intercorrelations among the study variables. We begin by noting briefly some observations regarding the personality variables. As with previous findings, the six HEXACO factor-level scales did not show strong intercorrelations, the highest ones being between Honesty–Humility and Agreeableness (0.28 in self-reports; 0.35 in observer reports). Self/observer correlations were relatively higher for Emotionality (0.53), Extraversion (0.58), and Openness to Experience (0.60) than for Honesty–Humility (0.44), Agreeableness (0.41), and Conscientiousness (0.41). As expected, the correlation between self-reported CN and CH was significant and moderately strong (0.44). The strongest self-report personality correlates of both CN and CH were Openness to Experience followed by Honesty–Humility, but in personality observer reports only Openness to Experience was substantially associated with CN and CH.

To investigate the relations of CN and CH and their hypothesized personality correlates, we tested a structural equation model. In this model, each construct was defined by a single scale score, and the uniqueness term of each indicator was fixed to a value obtained from the variance and internal-consistency (alpha) reliability of the scale score (i.e., variance × [1 – reliability]). Therefore the parameter estimates in the model represent the relationships among the latent constructs. The two identification variables (i.e., community and country) were included in the model as control variables.

**FIGURE 1:**
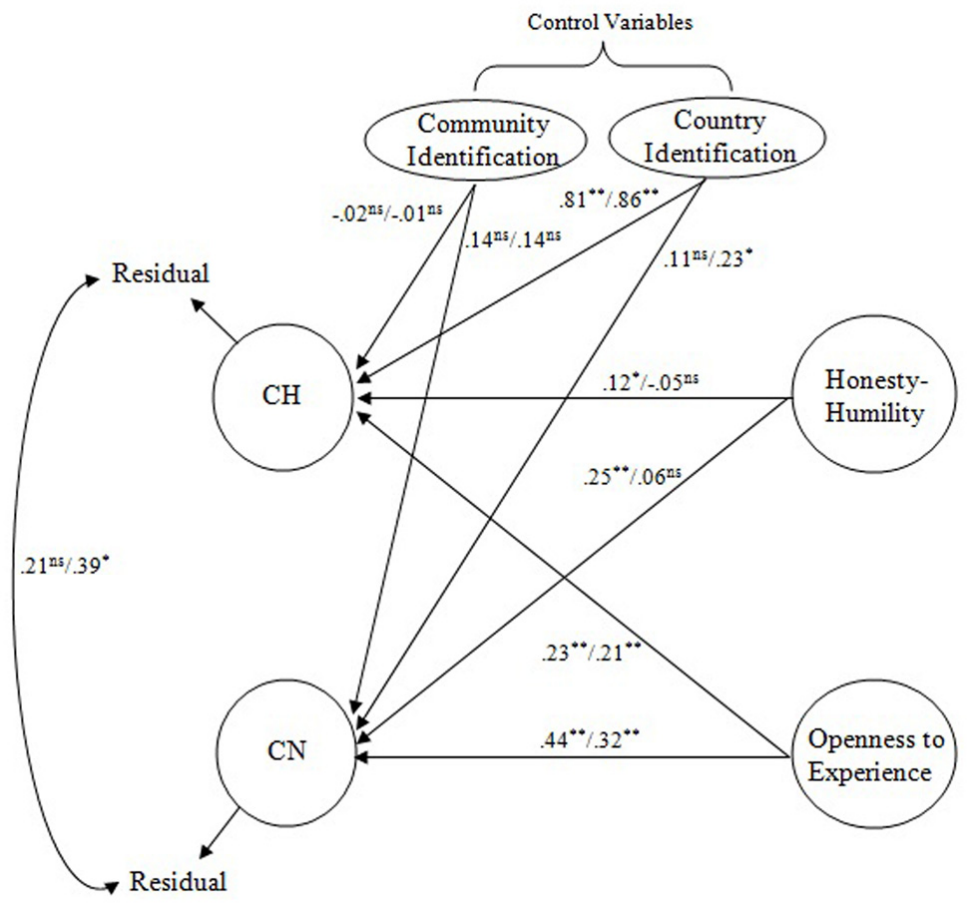
**A Saturated Model of CH, CN, Honesty–Humility and Openness to Experience.** CH, Connectedness to Humanity; CN, Connectedness to Nature; H, Honesty–Humility, O, Openness to Experience. Values on the left (right) side were standardized coefficients obtained from the model involving self-reported (observer reported) personality. ^∗^*p* < 0.05, ^∗∗^*p* < 0.01.

**Figure 1** summarizes the results of the structural equation analyses for the model. The values in **Figure 1** are standardized coefficients. The values on the left side of the slashes were obtained from the model involving self-reported personality, and the values on the right side from the model involving observer reported personality. Openness to Experience was found to show the strongest relations to both CH and CN (standardized coefficient = 0.23 and 0.44, respectively) and Honesty–Humility showed the second strongest coefficients to both CH and CN (standardized coefficient = 0.12 and 0.25, respectively). The hypothesis thus received strong support when personality variables were measured with self-reports. A *post hoc* exploratory analysis suggested that there were no other personality scales that showed significant associations with CH or CN. The latent correlation between CH and CN was 0.46 (after controlling for the two identification variables) when there was no path from personality, but the residual correlation between CH and CN observed in **Figure 1** (conceptually a partial correlation controlling for the personality and identification variables included in the model) was 0.21, which was marginally significant (*p* < 0.08). It appears that a fairly large portion of the correlation between CH and CN can be explained in terms of their common associations with Openness to Experience and Honesty–Humility. Nevertheless, a non-trivial portion of the relationship between CH and CN remained unexplained by the personality variables.

Regarding the model involving observer reported personality (i.e., values on the right side of the slashes in **Figure 1**), Openness to Experience again showed the strongest relations with CH and CN (standardized coefficient = 0.21 and 0.32 respectively). Honesty–Humility, however, did not show significant relationships with either CH or CN. These results might suggest that, given the somewhat weaker self/observer agreement for Honesty–Humility (*r* = 0.44) than for Openness to Experience (*r* = 0.60) in the present research, the strength of the relationship between observer reports of Honesty–Humility and self-reports of CN/CH may have been discounted to a stronger degree. Because the cross-source relationships between personality and CH/CN were relatively weaker than the corresponding within-source relationships, the residual correlations between CH and CN remained relatively large (*r* = 0.39) in this model. Finally, a *post hoc* analysis suggested that there were no other personality scales that significantly contributed to the prediction of CH or CN. (Only Emotionality showed a marginally significant contribution to the prediction to CN.)

**FIGURE 2:**
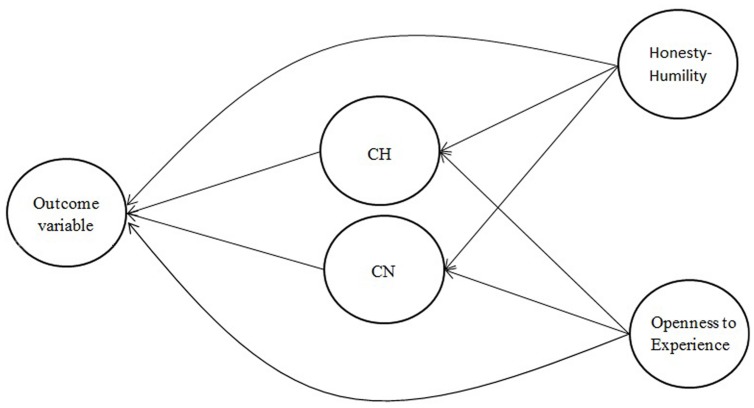
**A Graphical Depiction of the Mediation Analysis.** CH, Connectedness to Humanity; CN, Connectedness to Nature.

To examine the mediating roles played by CN and CH in the relations of the two personality variables (Honesty–Humility and Openness to Experience) with the three outcome variables (i.e., pro-environmental attitudes, pro-animal attitudes, and ecological behaviors), we expanded the above model three times, each time by adding one of the latter three variables but omitting the other two (see **Figure 2**). For these models, we also adopted the single indicator approach and included the two identification variables (i.e., community and country) as control variables as recommended by McFarland et al. (2012); however, the paths involving the control variables are not shown in **Figure 2** for the sake of simplicity. Each model was run twice, once using self-reports of personality and once using observer reports of personality. The total, indirect, and direct effects in the mediation models were tested for significance using the Bootstrap estimation procedure in Amos 20.

**Table 2** shows the results of the mediation analyses. With respect to pro-environmental attitudes as measured by the NEP, self- and observer reports of Openness to Experience showed significant total effects (0.371 and 0.332, *p* < 0.01), but only self-reports of Honesty–Humility were found to be marginally significant (0.152, *p* = 0.06). These three relationships were significantly mediated by the two mediating variables, but primarily through CN, rather than through CH. No significant direct effects were observed, suggesting that effects of both Honesty–Humility and Openness to Experience on NEP were primarily mediated through CN.

**Table 2:** Results of the mediation analyses.

	Environmental attitudes (NEP)		Ecological behavior		Animal Attitudes	
	Openness	Honesty-Humility	Openness	Honesty-Humility	Openness	Honesty–Humility
**Self-reported personality**
Total Effect	0.37^∗∗^	0.15^+^	0.38^∗∗^	0.19^∗^	0.08	0.29^∗∗^
Direct Effect	0.13	0.01	0.19	0.08	-0.27^∗^	0.10
Indirect Effect	0.24^∗^	0.14^∗^	0.20^∗^	0.11^∗^	0.35^∗∗^	0.20^∗∗^
Via CN	0.23^∗∗^	0.13^∗∗^	0.15^∗∗^	0.09^∗∗^	0.26^∗∗^	0.15^∗∗^
Via CH (IWAH)	0.02	0.02	0.05	0.02	0.09	0.04
**Observer reported personality**
Total Effect	0.33^∗∗^	0.02	0.37^∗∗^	0.16^∗^	-0.02	0.17^+^
Direct Effect	0.13	0.00	0.19	0.17^+^	-0.32^∗∗^	0.18^∗^
Indirect Effect	0.20^∗∗^	0.02	0.18^∗^	0.00	0.30^∗∗^	0.00
Via CN	0.17^∗∗^	0.02	0.12^∗∗^	0.02	0.19^∗∗^	0.03
Via CH (IWAH)	0.03	-0.01	0.07^∗∗^	-0.02	0.11^∗∗^	-0.03

*N = 321. CN, Connectedness to Nature; CH, Connectedness to Humanity as measured by the Identification with All Humanity (IWAH); NEP, New Environmental Paradigm. ^+^p < 0.10, ^∗^p < 0.05, ^∗∗^p < 0.01.*

Both self- and observer reports of Openness to Experience showed significant total effects on ecological behaviors (0.38 and 0.37, *p* < 0.01), and these effects were again primarily mediated more through CN than through CH (0.147 vs. 0.051 for self-report; 0.116 vs. 0.068 for observer reports), and no significant direct effects were observed. Both self- and observer reports of Honesty–Humility showed significant total effects on ecological behaviors (0.192 and 0.163, *p* < 0.05). Interestingly, while the indirect effect (but not the direct effects) was significant for self-reports of Honesty–Humility primarily via CN (0.110, *p* < 0.05), only the direct effect (but not the indirect effect) was marginally significant for observer reports (0.167, *p* = 0.06). The latter findings are due to the weak correlations of CH and CN with *observer reports* of Honesty–Humility, which nevertheless showed a moderate correlation with ecological behaviors. A similar finding was observed with respect to pro-animal attitudes (see below).

With regard to pro-animal attitudes, only self- and observer reports of Honesty–Humility showed significant total effects (0.294, *p* < 0.01 for self-reports; 0.173, *p* = 0.052 for observer reports). As with the findings involving ecological behaviors, only the indirect effect (not the direct effect) was significant for self-reports of Honesty–Humility, and the converse pattern was observed for observer report Honesty–Humility. Again observer reports of Honesty–Humility correlated more strongly with pro-animal attitudes than with the proposed mediating constructs (i.e., CH or CN). However, future research is needed to determine whether this result can be replicated in other samples.

Although neither self- nor observer reports of Openness to Experience showed significant total effects on pro-animal attitudes, both were found to have fairly strong indirect effects (0.353 and 0.300 for self- and observer reports respectively, *p* < 0.01). This occurred because there were significant direct effects in a *negative* direction (-0.272 and -0.320 for self- and observer reports respectively, *p* < 0.05). We speculate in Section “Discussion” on a possible reason for the negative direct effects.

## Discussion

The CH and CN constructs involve psychological oneness in relation to somewhat different entities (i.e., identification with humanity and identification with non-human living things). However, we found them to be significantly related to each other (latent *r* = 0.46, after controlling for identification with community and country). As hypothesized, both constructs shared Openness to Experience and (to a lesser degree) Honesty–Humility as their main personality correlates, and a substantial portion of the relationship between CN and CH could be explained by their common associations with these two personality traits. It appears that nature lovers are more likely to be believers in one human family, and that high levels of Honesty–Humility and (especially) Openness to Experience characterize such people.

Interestingly, though, the latent correlation between CN and CH was not fully explained by their common associations with these personality factors. Although additional research is needed to understand the meaning of this remaining covariation, we can speculate on some possibilities. First, CN and CH might be common manifestations of a construct representing people’s tendency to see fuzziness in the boundary between two entities. Consistent with this view, Hartmann’s (1991) concept of “boundary” (i.e., the permeability of mental boundaries) includes a facet called “opinions about peoples, nations, groups” which has considerable conceptual overlap with CH. Alternatively, CN, which closely resembles Wilson’s (1984) biophilic tendency (i.e., love of living things), may actually encompass CH. Specifically, the tendency to feel connected to living things would involve a connection not only to animals and plants, but also to persons from other ethnic, religious, or racial groups. Finally, CH might directly influence CN, to the extent that people who perceive some essential unity of humanity might carry that thinking over to the relations of humans with other living things.

We also investigated the mediating roles of CN and CH in the relations of Honesty–Humility and Openness to Experience with pro-environmental/pro-animal attitudes and ecological behaviors. In general, CN was a more important mediator than was CH. The direct effects of Honesty–Humility and Openness to Experience on pro-environmental attitudes (i.e., NEP) and ecological behaviors were weak, which suggests that CN explains most of the association of these two personality variables with pro-environmental attitudes and ecological behaviors.

It was also found that Openness to Experience has a significant *positive* indirect effect on pro-animal attitudes through CN and CH, but that its zero-order correlation with pro-animal attitudes was very weak, suggesting the existence of suppression variables. This observation was robustly found across self- and observer ratings of Openness to Experience. A careful perusal of Herzog et al.’s (1991) Animal Attitudes Scale indicates that some items concern attitudes about the use of animals for food and in medical or scientific research, whereas other items concern attitudes about animal welfare in general (or about use of animals for recreational purposes such as entertainment and hunting). Openness to Experience tended to correlate positively with pro-animal attitudes captured by the latter items, but it showed near-zero or even negative correlations with pro-animal attitudes as captured by the former items. That is, while people high in Openness to Experience have generally sympathetic attitudes toward animals, with these attitudes being attributable to their high levels of CH and CN, those people might still support animal use for medical research and scientific advancement. As such, adopting an affection-oriented measure of animals attitudes (e.g., the love of animal scale, Ray, 1982) might produce a somewhat different result from what was observed in the present research.

The results of the present analyses confirmed that Openness to Experience is the primary personality correlate of pro-environmental attitudes and behaviors (Markowitz et al., 2012), and also indicated that Honesty–Humility is positively associated with pro-environmentalism, which supports the theoretical reasoning discussed by Hilbig et al. (2013). The present findings shed some light on the mixed findings regarding the roles of prosocial personality traits in influencing pro-environmental behaviors and attitudes. As discussed in Section “Introduction,” Markowitz et al. (2012) reported results from US samples showing that prosocial personality traits such as Honesty–Humility and Big Five Agreeableness were not linked to behaviors and attitudes regarding pro-environmentalism or to indicators of CN (see also Leary et al., 2008). In contrast, other studies conducted in Europe, in Canada, or in Hong Kong (Hirsh and Dolderman, 2007; Swami et al., 2011; Hilbig et al., 2013; Tam, 2013) have reported moderately strong relations of prosocial personality variables (e.g., Honesty–Humility, low Machiavellianism, and Big Five Agreeableness) with variables related to environmentalism.

Hilbig et al. (2013) described two possible sources for the difference between the results of Markowitz et al. (2012) and those of the other studies: (1) different measures of ecological behaviors used in each study, and (2) different perceptions about environmentalism across countries. In the present study, the Honesty–Humility factor and CH were found to correlate positively with CN (0.32 and 0.44), NEP (0.16 and 0.14), and ecological behaviors (0.21 and 0.27). Given that the environmentalism variables included in the present research are identical or very similar to those included in Markowitz et al. (2010; e.g., CNS and NEP), the present findings might rule out the first explanation for the discrepant results. In addition, a recent study conducted in the US (Brick and Lewis, in press) also reported significant positive associations between Honesty–Humility and pro-environmental behaviors, suggesting that the mixed findings may not be due to the differences across the countries. Although more studies are needed to clarify the ambiguity present in the literature, the results so far appear to suggest that prosocial personality traits are likely to be implicated in pro-environmental attitudes and behaviors.

The positive links of Honesty–Humility and Openness to Experience with pro-environmental variables support some important notions discussed in the Value-Belief-Norm (VBN) theory of environmentalism proposed by Stern and Dietz (1994) and Stern (2000). According to that theory, there are broadly three value bases for environmentalism, which include egoistic, altruistic, and biospheric value orientations. That is, people’s approaches to various environmental issues are based on perceived costs and benefits for themselves (egoistic), for other people (altruistic), or for nature itself (biospheric). The two personality variables shown to predict variables related to environmentalism are closely associated with some of these values. Specifically, Honesty–Humility is aligned well to altruistic values and Openness to Experience to biospheric values.

Moreover, the altruistic value orientation corresponds to one of the two basic value dimensions (Schwartz, 1992), namely, the Self-transcendence (vs. Self-enhancement) dimension, and the biospheric value orientation corresponds to some aspects of the other value dimension, namely Openness to Change (vs. Conservation). These two value dimensions have also been found to predict environmentally significant behaviors (Karp, 1996). We should also note that Honesty–Humility and Openness to Experience have been suggested and found to be the two personality bases for the basic value dimensions (Lee et al., 2009, 2010). As such, the altruistic and biospheric value orientations in the VBN theory are theoretically and empirically linked to Honesty–Humility and Openness to Experience.

The results of the present and previous research suggest some practical implications for efforts to promote environmentally friendly behavior. First, the recurrent finding that Openness to Experience is positively associated with pro-environmental behavior suggests that one challenge will be to encourage that behavior in persons who are low in Openness. However, we expect that as environmentally friendly behaviors become more widespread, they will no longer be seen as unconventional, and this may diminish any reluctance by low-Openness persons to engage in them. A second implication follows from the (less consistent) finding of a positive link between Honesty–Humility and pro-environmental behavior: to the extent that persons low in Honesty–Humility are intrinsically less inclined to engage in such prosocial activity, one plausible way to promote it would be to institute monetary incentives.

One advantage of the present research is that both self- and observer ratings of personality were used. In the past, most studies relied on the same rating source for personality and pro-environmental variables, and the effect sizes observed in these studies might have been influenced by same-source specific variances. In the present research, the findings involving Openness to Experience were generally robust across the two rating sources. In contrast, observer reports of Honesty–Humility tended to show noticeably weaker correlations with other variables—except ecological behaviors and animal attitudes—than did self-report of Honesty–Humility. It is uncertain whether this indicates inflated effect sizes in the same-source dataset, or somewhat limited validity of observer rated Honesty–Humility in the present research (self/observer agreement of Honesty–Humility in the present research was 0.44 and the corresponding figure for Openness was 0.60). Future research clarifying this issue is in order.

We should mention some noteworthy findings related to the IWAH scale of McFarland et al. (2012), which was used to operationalize CH in the present research. The IWAH scale also provides measures of identification with one’s community and identification with one’s country, and McFarland et al. (2012) suggested that these two identification variables may need to be controlled to examine the characteristics that are uniquely associated with IWAH. In the present research, by controlling for the two identification variables, we were able to examine a purer measure of CH, and this distinguishes the present research from other work examining general connection to other people (e.g., Leary et al., 2008). Another interesting aspect of the results related to McFarland et al.’s (2012) scales is their pattern of correlations with Openness to Experience. The three identification variables showed a sharply linear pattern of correlations with Openness to Experience as the boundary of the identification object gets larger. That is, self-reports of Openness to Experience correlated 0.06, 0.23, and 0.39 with the three identification variables in the order of community, country, and all humanity, and observer reports of Openness to Experience correlated 0.00, 0.13, and 0.27 with the same variables (see **Table 1**). This finding does seem to suggest that high Openness is one of the distinguishing characteristics of the people who have a fuzzier distinction between ingroups and outgroups, and provides additional construct validity evidence for IWAH.

Finally, we should note that the present data are cross-sectional, and do not allow us to make any causal inferences. Nevertheless, we believe that personality variables are more “fundamental” than the other variables examined in the present research. The basic personality dimensions represent tendencies of action, thought, and feeling that are general across contexts, whereas the CH and CN constructs are much more specific. Therefore, we presume that the personality factors causally precede CH and CN, which represent attitudes toward specific objects.

## Summary

CH and CN involve people’s orientations toward two superficially different entities—the human race and the natural world. Nevertheless, these constructs correlate positively with each other and share the Openness to Experience and Honesty–Humility factors as their primary personality correlates. In addition, pro-environmental and pro-animal attitudes and behaviors also showed significant relations with the same personality dimensions, and these relationships were primarily mediated by CN and to a lesser degree by CH.

## Conflict of Interest Statement

The authors declare that the research was conducted in the absence of any commercial or financial relationships that could be construed as a potential conflict of interest.
